# Sentiment analysis of political communication: combining a dictionary approach with crowdcoding

**DOI:** 10.1007/s11135-016-0412-4

**Published:** 2016-09-21

**Authors:** Martin Haselmayer, Marcelo Jenny

**Affiliations:** Department of Government, University of Vienna, Rooseveltplatz 3/1, 1090 Vienna, Austria

**Keywords:** Sentiment analysis, Crowdcoding, Political communication, Negative campaigning, Media negativity

## Abstract

Sentiment is important in studies of news values, public opinion, negative campaigning or political polarization and an explosive expansion of digital textual data and fast progress in automated text analysis provide vast opportunities for innovative social science research. Unfortunately, tools currently available for automated sentiment analysis are mostly restricted to English texts and require considerable contextual adaption to produce valid results. We present a procedure for collecting fine-grained sentiment scores through crowdcoding to build a negative sentiment dictionary in a language and for a domain of choice. The dictionary enables the analysis of large text corpora that resource-intensive hand-coding struggles to cope with. We calculate the tonality of sentences from dictionary words and we validate these estimates with results from manual coding. The results show that the crowdbased dictionary provides efficient and valid measurement of sentiment. Empirical examples illustrate its use by analyzing the tonality of party statements and media reports.

## Introduction

Sentiment analysis of textual data has manifold applications in the social sciences, among them the study of polarization, public opinion or media tone (e.g., Monroe et al. 2008; Van Atteveldt et al. 2008; Hopkins and King 2010; Soroka 2012; Young and Soroka 2012; Burscher et al. 2015; González-Bailón and Paltoglou 2015; Soroka et al. 2015a, b). However, a lack of tools or procedures for producing or collecting sentiments ratings of acceptable quality for large-scale data analyses currently hampers progress, in some languages more than in others.

 Computer-based approaches dominate the field of sentiment analysis, which attempt to produce the same sentiment rating of texts as a human reader. Unfortunately for social scientists interested in phenomena such as political polarization or media tone in non-English countries, automated methods exhibit a strong language bias as they are developed and validated predominantly with textual data in English language. The number of sentiment analysis tools available for other languages is much smaller and their output tends to be of lower quality (Mohammad 2016).

If computer-based sentiment analysis is not available or its results are not good enough, one can resort to traditional content analysis with human coders. However, in ‘big data’ research projects manual content analysis quickly faces the restrictions of limited time, money and small numbers of trained coders.

We outline a measurement procedure that (1) alleviates resource constraints (2) produces sentiment ratings that meet conventional quality standards, and (3) allows a researcher to conduct sentiment analyses in his or her language and domain of interest. Applying this procedure, we create a German language sentiment dictionary for the analyses of party statements and media reports. We use crowdcoding, the services of online coders, to produce the sentiment ratings of dictionary words. The sentiment dictionary is available for download,^1^ but similar to Laver and Garry (2000, p. 626) we want to highlight the procedure rather than a specific product: “Most important, given changing political meanings of words over time and space, is the *procedure* for deriving a dictionary, rather than the substantive content of any given dictionary.” By presenting our procedure we want to support “sentiment analysis in the resource poor languages” (Mohammad 2016, p. 203) and encourage the creation of customized dictionaries that fit well for the domain (Grimmer and Stewart 2013) *and* language studied.

The structure of the paper is as follows. The next section deals with the measurement of sentiment in political discourse. Section three introduces crowdcoding as a data collection technique. Section four covers the creation of a sentiment dictionary, section five the rating of texts. Then we compare dictionary-based sentiment scores of texts with the results of manual coding. We also compare the scores from our custom-built sentiment dictionary for political communication with scores from existing, non-domain specific sentiment dictionaries. Section six includes two empirical illustrations to show the value of the data produced. The first covers negative campaigning in the 2013 Austrian national elections, the second looks into media tone. Finally, we discuss critical points and outline future uses of the procedure.

## Measuring sentiment in political texts

Sentiment analysis measures the polarity or tonality of texts by identifying and assessing expressions people use to evaluate or appraise persons, entities or events (Pang and Lee 2008; Liu 2012; Soroka 2014; Mohammad 2016). Analyzing the polarity of texts has a long tradition in the social sciences. A prominent example is media negativity, a concept that captures the over-selection of negative over positive news, the tonality of media stories, and the degree of conflict or confrontation in the news (Esser and Strömbäck 2012). Its “measurement in quantitative content analytic research can be defined as the process of linking certain aspects of textual data to numerical values that represent the presence, intensity, and frequency of textual aspects relevant to communication research” (Lengauer et al. 2012, p. 183). A number of recent studies demonstrate the benefits of sentiment analysis for such analyses (Van Atteveldt et al. 2008; Soroka 2012; Young and Soroka 2012; Burscher et al. 2015; Soroka et al. 2015a, b). Sentiment analysis has also been used to establish the level of support for legislative proposals or polarization from the analysis of parliamentary debates (Monroe et al. 2008), to identify issue positions or public opinion in online debates (Hopkins and King 2010; Ceron et al. 2012; González-Bailón and Paltoglou 2015), or for studying negative campaigning (Kahn and Kenney 2004; Lau and Pomper 2004; Geer 2006; Nai and Walter 2015) to mention just a few prominent uses. The classification of text as positive, negative, or neutral, is denoted by expressions such as polarity, valence or tone (Wilson et al. 2005; Young and Soroka 2012; Thelwall and Buckley 2013; González-Bailón and Paltoglou 2015; Mohammad 2016). An incomplete list of terms for the gradual or quantitative measurement of sentiment includes potency (Osgood et al. 1957); intensity, sentiment strength (e.g. Thelwall et al. 2010) or emotive force (Macagno and Walton 2014). We will use sentiment strength and tonality as synonymous terms for a fine-grained measure of negativity. We cover only the neutral to negative part of the sentiment scale as psychological research has highlighted asymmetries between positive and negative evaluations of situations, persons or events (Peeters 1971; Peeters and Czapinski 1990; Cacioppo and Berntson 1994; Baumeister et al. 2001; Rozin and Royzman 2001). We also do not probe into different ‘negative’ emotions (Ekman 1992) nor look at causes of negative evaluations (Soroka 2014; Soroka et al. 2015a).

The field of sentiment analysis is dominated by computer-based, automated approaches whose progress varies strongly by language (Mohammad 2016). Many social scientists will be still more familiar with human-based content analyses with or without dictionaries (Stone et al. 1966; Budge and Farlie 1983; Baumgartner and Jones 1993; Laver and Garry 2000; Young and Soroka 2012; Krippendorff 2013). Both manual and automated text analysis require an initial step of coding (or annotating or labelling) the sentiment of a text unit. Supervised and non-supervised automated approaches employ sample texts with coded sentiment ratings to ‘learn’ the sentiment of words. Once that phase of the research process has concluded—which usually includes a considerable amount of ‘fine-tuning’ the procedure—, the algorithms are scalable to large text corpora. Manual coding, in contrast, does not scale well as human coders often have to rate small units of texts such as sentences or words. Compared to unit by unit hand coding creating and using a dictionary of words already coded is a big step towards higher efficiency. An automated search can then find out whether a new text unit contains a dictionary word and retrieve its sentiment value.

A basic assumption of using a dictionary is that it contains the most important words required for rating a text. A recent comparison of English language dictionaries and machine learning approaches found that “dictionaries had exceptional precision, but very low recall, suggesting that the method can be accurate, but that current lexicons are lacking scope. Machine learning systems worked in the opposite manner, exhibiting greater coverage but more error” (Soroka et al. 2015a, p. 112). A large dictionary can provide good scope, but dictionary size on its own misleads about the quality of the output as irrelevant vocabulary produces less discriminating sentiment scores (González-Bailón and Paltoglou 2015).

 Related is the problem of domain specificity. Sentiment scores of words extracted from a training set of annotated texts do not generalize well to texts from other domains. Social scientists have accordingly stressed the need for custom-made dictionaries (Loughran and McDonald 2011; Young and Soroka 2012; Grimmer and Stewart 2013; González-Bailón and Paltoglou 2015; Soroka et al. 2015a). Even some commercial providers advise against using a sentiment dictionary ‘as is’ without thorough customization.^2^

We have pointed out that creating a customized dictionary or setting up a sample of training texts for machine learning requires an initial step of human coding which will be a procedural bottleneck if unit-by-unit sentiment coding has to be done with a small number of coders. We mitigate this bottleneck through crowdcoding, which offers a cheap and fast way to collect annotations for large amounts of text.

## Employing crowdcoding^3^ to create a sentiment dictionary

The idea of crowdsourcing draws on “wisdom of the crowd” arguments (e.g., List and Goodin 2001) and evaluations of expert-coded versus crowdcoded data show that for many tasks small aggregates of non-expert annotations are as good as single-expert annotation (Snow et al. 2008; Alonso and Baeza-Yates 2011).

Crowdsourcing online platforms such as Amazon’s Mechanical Turk, CrowdFlower and others provide access to an international large workforce for “micro tasks” requiring human intelligence. These lay coders have identified sentiment in texts with good results (e.g., Hsue et al. 2009; Taboada et al. 2011). Political scientists have employed crowdsourcing for data generation (Berinsky et al. 2012, 2014), for instance for content analyses of election manifestos (Benoit et al. 2016).

Using a large anonymous online workforce naturally raises data quality concerns. The best crowdsourcing platforms provide tools for quality control and real-time scrutiny of the data generation process, such as coder recruitment based on previous work record, skills, context knowledge or geographic location. Test questions can be randomly interspersed in a coding task to identify bad performance, and “screener” questions to check the attention of coders during the coding process (Berinsky et al. 2014).

Crowdcoding facilitates the completion of a large coding project at relatively low costs. Yet it still is unit by unit coding, and time and monetary costs increase with the number of units to be coded. If the goal is to code large amounts of texts using a dictionary with a good scope is an economic alternative. Creating the dictionary is a one-time, fixed-coast investment in time and money. Its application to large text corpora incurs few additional costs, apart from some text preprocessing. Large-scale text analyses can be easily repeated whenever a dictionary gets additional entries or modified sentiment scores. The next section shows step by step how to build your own sentiment dictionary.

## Building a negative sentiment dictionary

A negative sentiment dictionary consists of words with sentiment scores. Our procedure contains the following steps:

1. Sampling sentences from the domain of interest
2. Crowdcoding the sentiment strength of sentences
3. Estimating a sentence tonality score
4. Estimating a word tonality score
5. Discriminating between important and unimportant words

Note that we move from words to sentences and back to words as relevant units. The reason is that we ask coders to rate complete sentences instead of single words taken out of context.

**Fig. 1:**
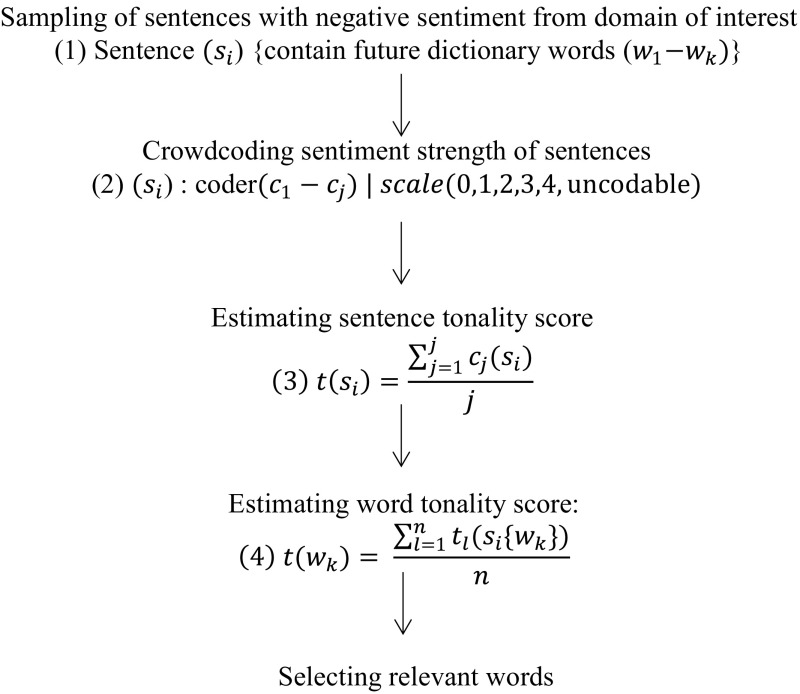
Creating a sentiment dictionary*. Notes* i…number of sentences, j…number of coders, k…number of dictionary words, l…number of tonality ratings, n…number of sentences containing a rated word

Figure 1 shows a flow chart of the procedure:

### Sampling sentences with negative sentiment from domain of interest

We are substantively interested in the tonality of political communication of Austrian parties and media, and assemble a corpus of party press releases, minutes of parliamentary debates and media reports on election campaigns from the years 1995–2013. The texts are available in machine-readable format.^4^

Following Remus et al. (2010) and Liu (2012) we use a small set of common negative words from existing German language sentiment dictionaries as “seed words” to select potentially negative sentences (Remus et al. 2010; Waltinger 2010; Momtazi 2012; Diwisch and Siegel 2014). The corpus initially consists of about 470,000 sentences. Pre-filtering with seed words cuts its size to about 215,000 sentences with negative sentiment. From that corpus, we randomly select 13,000 sentences for crowdcoding.^5^ Pre-filtering with seed words is not required, but it reduces the coding costs. The alternative is to submit an unfiltered, random sample of sentences to crowdcoding, with many of these subsequently coded as neutral or positive. While this strategy will increase the coding costs, some may view the collecting of negative, positive and neutral sentiment words as an advantage.

### Crowdcoding the sentiment strength of sentences

As the texts are in German, we recruit only coders from Austria or Germany through the platform CrowdFlower. We provide the coding instructions in the Appendix. Each sentence is assigned to ten coders to rate its negativity on a 5-point scale ranging from 0 (not negative) to 4 (very strongly negative) or judge it uncodable. Individual coder performance is monitored. Before the actual coding begins four test questions have to be answered correctly, and one out of five sentences of a task is another test question.

The selection of appropriate test sentences is crucial for the successful application of crowdsourcing. We (i) first selected a large number of sentences that we judged unanimously, (ii) asked a group of ten colleagues and student assistants to code them too, and (iii) selected only those that showed very strong agreement in the tonality coding. We also pretested the coding jobs to collect feedback from the crowd. After completion, coders evaluate a job, including the quality of the instructions and the fairness of test tasks. Finally, it is possible to monitor the performance of test questions in real time and to remove or adapt them if problems occur. Coding sentiment strength on a five-point ordinal scale is difficult (Pang et al. 2002; Hopkins and King 2010) and for the test questions we accepted two adjacent options on the five-point scale as correct answers. Coders “usually have difficulty distinguishing between two adjacent ordinal classes whereas distinguishing between two classes which are far away from each other is much easier” (Zhou et al. 2014, p. 2). The probability of passing the first test by guessing is only 4 % and it gets smaller with each additional test unit.^6^ A coder dropping below an accuracy threshold of 75 % during coding is stopped from further contributing and his or her data not included in the data set. 480 coders answered on average 92 % (standard deviation of 0.07) of the tests questions correctly and contributed ratings to the data set. Overall, we collected about 130,000 valid codings (ten ratings per sentence), split into eight tasks, for which we paid 2000 Euros in total (about 2200 US-dollars) through the crowdcoding platform. We provide a list of the crowdcoding jobs in the Appendix.

### Estimating sentence tonality scores

For each sentence we collect negativity ratings from ten coders (*c*_*j*_) and calculate a mean sentence score *t*(*s*_*i*_).

1 $$t(s_{i} ) = \frac{{\sum\nolimits_{j = 1}^{j} {c_{j} (s_{i} )} }}{j}$$

### Estimating word tonality scores

This is also the initial tonality score of each word or more specifically word form contained in a sentence. We restrict the dictionary to single words and do not consider combinations of words (bigrams, trigrams) or short phrases. We lemmatize the word forms and do part-of-speech tagging with the tool Treetagger (Schmidt 1994), a process that due to the current quality of such tools for the German language requires some manual post-processing of results. An alternative to lemmatizing is to include all existing word forms in the dictionary (e.g. Remus et al. 2010), which is difficult with languages, such as Finnish (or Hungarian, Turkish, and Russian), where nouns can take up to 2000 different forms. Here, a feasable strategy is to concentrate on the most important word forms (Kettunen and Airio 2006). Then we check the frequency of words. If a word *w*_*k*_ appears in more than one sentence, we calculate a mean word score from these sentences *t*(*s*_*i*_).

2 $$t(w_{k} ) = \frac{{\sum\nolimits_{l = 1}^{n} {t_{l} (s_{i} )} }}{nk}$$

Note that the double step of mean aggregation of ordinal scores produces numbers with decimal places, which methodological purists can object to. Alternative aggregation measures exist if the goal is to preserve the original 5-point scale (Dawid and Skene 1979; Zhou et al. 2014; Felt et al. 2015). A recent crowdsourcing study by Benoit et al. (2016) found that “means of means” gave almost the same results as more complex algorithms.

### Separating relevant and irrelevant words

At this stage the complete list or “bag of words” contained in the rated sample of sentences has a sentiment score. However, we want only words in a sentiment dictionary that express negative tonality with a high probability and delete the rest of the list as irrelevant. We start by cleaning the database and remove all words that have less than three characters (n = 960, most of which are due to errors in text pre-processing). Word frequency is a standard indicator of relevance in automated text analyses. The more common a word is the less informative is it about a specific quality such as negativity. There is no gold standard for deleting high-frequency words. We delete words such as articles, pronouns as well as names using part-of-speech-tags (Schmidt 1994) and use stop word lists that identify highly frequent words based on the Leipzig Corpora Collection (Quasthoff et al. 2006) (n = 4518). For a different reason we also delete rare words. We aim at collecting word negativity as a global rather than as a local, highly context-dependent quality. Therefore, we drop unique words (n = 24,511) that appear in a single sentence as containing too much measurement error. We identify and remove positive words from existing sentiment dictionaries (Wolf et al. 2008; Klenner et al. 2010; Remus et al. 2010; Waltinger 2010; Momtazi 2012; Diwisch and Siegel 2014) (n = 3725). Then we delete any remaining named entities from the list. We use online available lists for named entity recognition (Faruqi and Pado 2010; Steinberger Ralf et al. 2011; Benikova et al. 2014) and a set of named entities from the AUTNES project to identify and delete the names of politicians, parties, or organizations (n = 6378). The deletion of named entities, of stop words and rare words reduces the number of words from initially about 40,000 to about 5000 words.

**Fig. 2:**
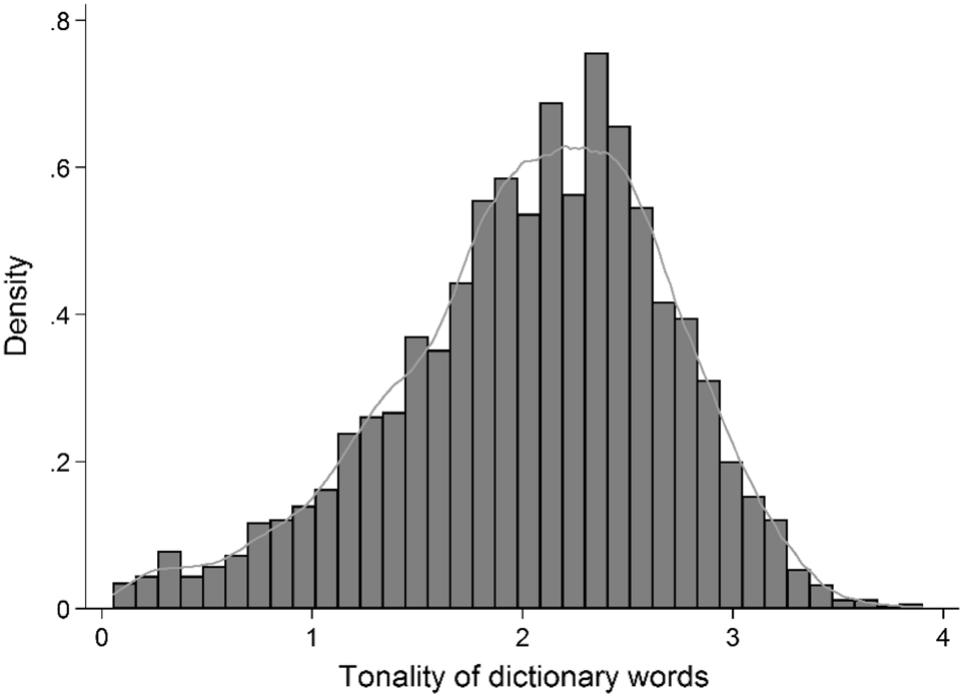
Histogram of tonality scores of dictionary words (n = 5001)

Figure 2 shows the distribution of tonality scores for the 5001 words in the dictionary which range from 0.06 to 3.8 on the scale from 0 (not negative) to 4 (very strongly negative). The mean tonality score of the dictionary words is 2.04 (standard deviation of 0.65).

## Scoring sentences and texts

Unit scale in sentiment analysis varies from documents to sentences to smaller textual unit such as word groups or single words. If a procedure estimates sentiment scores for words, one needs an aggregation rule to get a sentence-level or document-level score. Our scoring approach rests on the “bag of words” assumption (Laver et al. 2003; Monroe et al. 2008; Slapin and Proksch 2008). We equate the tonality of a sentence with the tonality of a dictionary word contained in it. If a sentence (*s*_*i*_) contains several different sentiment words (*w*_*k*_), we apply the “maximum” rule of Thelwall and Buckley (2013) which means the most strongly negative word (max(*w*_*k*_)) sets the tonality of the sentence.^7^

3 $$t\left( {s_{i} } \right) = { \hbox{max} }(w_{k} )$$

The dictionary includes negation words (n = 13, such as no, not, never, neither, without) and intensifier words (n = 53, e.g. completely, exceedingly, extremely, heavily, very). If a negation word immediately precedes a dictionary word, we exclude the latter from the calculation of the tonality score for the sentence rather than flipping the polarity of a sentence (see Thelwall et al. 2012). One could use a similar strategy for the negation of positive words, but we focus on negativity only. Taboada (2016, pp. 332–335) points out that polarity inversion usually translates into low intensity scores. Following Taboada et al. (2011), we amplify a dictionary word’s negativity score (by a factor of 1.25) if it is preceded by an intensifier word, up to the maximum value set by the scale’s boundary.

## Validating the procedure

Face validity (e.g., Monroe et al. 2008) and cross-validation (e.g., Laver et al. 2003; Slapin and Proksch 2008) are popular standards used to evaluate results from automated text analyses, but the gold standard is a comparison with results from human coding (Grimmer and Stewart 2013; Lowe and Benoit 2013, p. 13).

**Fig. 3:**
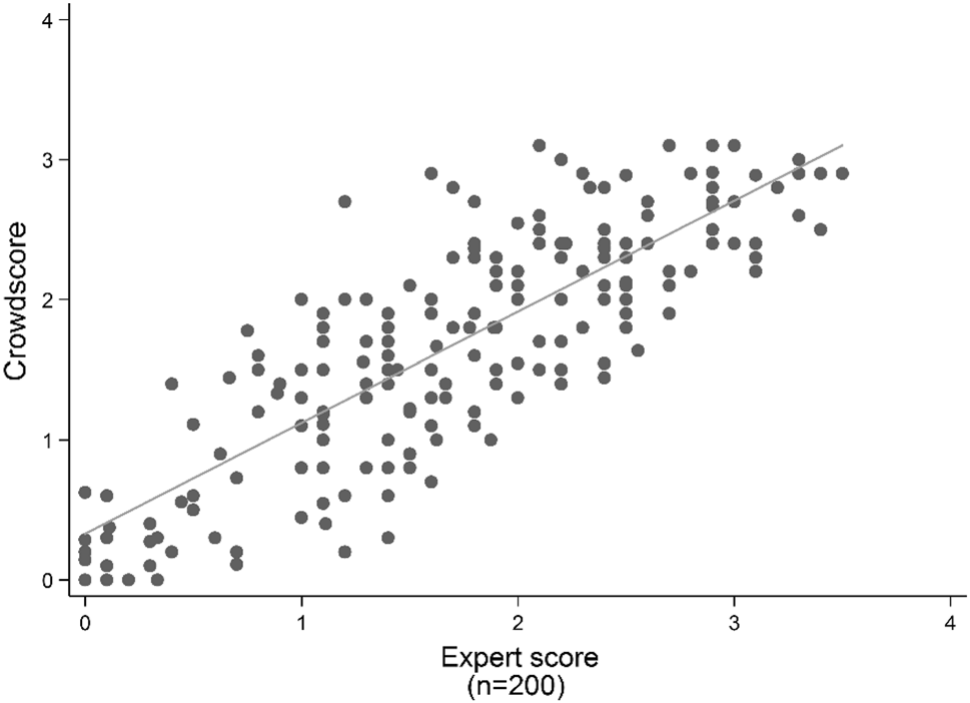
Comparing expert scores and crowdscores. *Note*: *Line* indicates linear regression of crowdscores on expert scores

 To check the validity of our approach we use a random sample of 200 sentences from party press releases from four national election campaigns held between 2002 and 2013 as well as media reports from the most recent campaign. Like Benoit et al. (2016), we evaluate the validity of our approach by comparing the aggregated, rather than individual coder results obtained through crowdcoding to manual expert annotation. Each of the authors separately coded the sample sentences on a 5-point scale. A group of ten online recruited coders completed the same task. The mean sentence scores, aggregated for two expert ratings on the one hand and the group of lay coders on the other hand, exhibits a Pearson correlation of 0.82 (Fig. 3). Thus in line with previous research, we find that the group of lay coders was able to replicate the expert data (e.g. Snow et al. 2008; Benoit et al. 2016), with a slight centrist bias in these aggregate ratings (Saal et al. 1980).

**Fig. 4:**
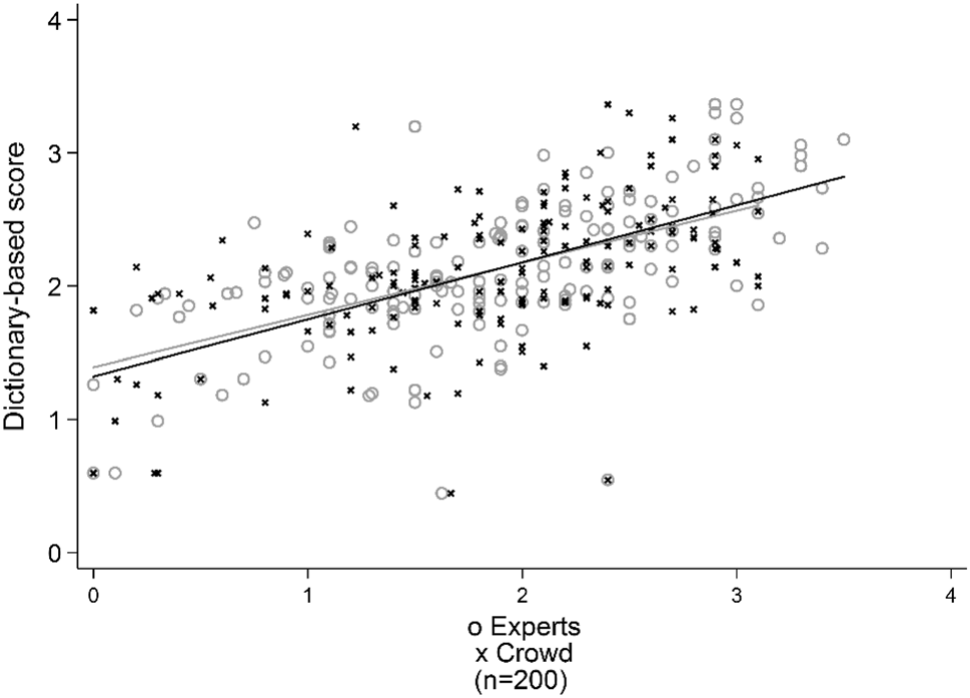
Comparing expert scores, crowdscores and automated, dictionary-based scores. *Note*: *Lines* indicate linear regression of dictionary-based scores on expert scores (*grey line*) and crowdscores (*black line*)

We score the test sample with our dictionary, which results in a Pearson correlation between manual expert ratings and dictionary ratings of 0.65 with 84 % coverage. The level of correlation is at par with recent English language sentiment analyses employing similar levels of granularity (e.g., Strapparava and Mihalcea 1556; Thelwall and Buckley 2013). Figure 4 provides a graphical representation of the correlation between dictionary-based scores and expert scores, including the linear regression between these two.^8^ There is again some degree of a centrist bias of the crowdscores due to mean aggregation.

To provide a direct test of our argument that we need context-sensitive dictionaries, we subject our sample to analysis with two other German-language sentiment dictionaries (Remus et al. 2010; Momtazi 2012). These dictionaries are translations of English sentiment dictionaries.^9^ Table 1 shows how their size and type of entries differs (e.g. words, word stems, word forms (including conjugation/declination), lemmas and synonyms). Applied to our test sample they exhibit a lower rate of coverage and their sentence scores have almost no correlation with the manual expert coding of negative sentiment strength. This confirms the point that dictionary-based analysis requires a customized dictionary to begin with (Grimmer and Stewart 2013; González-Bailón and Paltoglou 2015; Mohammad 2016).

**Table 1:** Characteristics of three German sentiment dictionaries, coverage and Pearson correlations with expert coding (n = 200 sentences). *Source* Remus et al. (2010), Momtazi (2012)

	Unique words	Search terms	Sentiment scale	Correlation with expert coding	Coverage (% matched sentences)
Political Sentiment Dictionary	5001	5001	0–4	0.65	84
Momtazi dictionary	1074	1074	0–4	0.13	46
Senti-WS dictionary	1818	13,814	0–1	0.19	31

Entries for the Senti-WS and Momtazi dictionary refer to the number of negative words in the dictionary

We check whether our dictionary is large enough to provide a good coverage of the phenomenon under study. A dictionary with perfect scope should assign a negativity score to all sentences with negative content. Accordingly, the 32 sentences out of 200 without a matching dictionary word should not be negative. We assign these sentences negativity scores of zero and recalculate the correlation with manual coding which results in a slightly lower Pearson correlation of 0.6. Closer inspection shows a few sentences without a dictionary word that coders rated negatively. Most of them contain separable verbs (that we could not match) or express irony or sarcasm. A few sentences are rhetorical questions. A politician’s statement *“Politics for Women is different”* expressed dissatisfaction without using a manifestly negative word. The rhetorical question *“Where has the Green’s objective environmental policy gone?”* criticizes the party’s actions without a negative word. Irony, sarcasm and rhetorical questions are common pitfalls in automated text analyses. However, as long as these phenomena do not make up a major portion of the text corpus, the coverage rate of our dictionary appears fine. It is at a par with comparable English language sentiment dictionaries (e.g. Strapparava and Mihalcea 2008; Thelwall and Buckley 2013).

## Applications

We now use the political sentiment dictionary in two applications. Specifically, we study the parties’ use negative campaigning and the tone of media coverage with data from the Austrian National Election Study.

### Negative campaigning in the Austrian national elections 2013

Research on negative campaigning has predominantly relied on binary classifications of statements as negative or non-negative (e.g., Damore 2002; Lau and Pomper 2004; Walter 2014) which is easier to operationalize than a fine-grained measure of tonality. However, we contend that the degree of negativity matters. Weak expressions of criticism have different effects than strongly worded attacks. Studies find that voters react to the intensity of negative messages (e.g., Mutz and Reeves 2005; Fridkin and Kenney 2011).

Negative campaigning featured prominently in the 2013 Austrian national elections. We analyze rhetorical interaction between parties via press releases during the final 6 weeks of the campaign. As part of the Austrian National Election Study (Müller et al. 2014), a relational content analysis with human coders of the headlines of 1958 party press releases was done. We use a subset of these press releases, which contain 755 directed, negative relations between two parties. Words from the sentiment dictionary matched 82 % of these statements. Table 2 shows the frequency and tonality of negative campaigning of the six parliamentary parties competing in the 2013 Austrian election.

**Table 2:** Amount and tonality of negative statements in party press releases. *Source* Own calculations based on AUTNES coding of 2013 national election campaign

Party	Negative statements		Statements with a tonality score		Mean tonality of statements
	Count	Percent	Count	Percent	
Social Democratic Party (SPÖ)	164	21.7	128	20.7	2.13
People’s Party (ÖVP)	199	26.4	169	27.3	2.36
Freedom Party (FPÖ)	232	30.7	193	31.2	2.24
Greens (Grüne)	59	7.8	53	8.6	2.16
Alliance for the Future of Austria (BZÖ)	68	9.0	48	7.7	2.05
Team Stronach (TS)	33	4.4	28	4.5	2.32
Total	755	100	612	100	2.23

The number of negative press releases sets the three largest parliamentary parties apart from the three smaller parties. The government parties SPÖ and ÖVP, and the opposition party FPÖ account for almost four out of five negative press releases. Studies of negative campaigning in multi-party systems argue that government parties use fewer negative campaign statements than opposition parties (e.g., Walter and Van der Brug 2013). At the same time, parties in government are expected to be the most important targets of negative campaigning (Walter 2014). We draw on these arguments to explore the patterns of negative campaigning in the 2013 election campaigns. Additionally, we want to test evidence from a recent study, that the government parties (SPÖ and ÖVP) devote most of their negative campaigning on each other (Dolezal et al. 2015). We expect that coalition partners criticize each other frequently but less strongly than other parties.

To test these expectations, we perform an OLS regression using the tonality of a press release as our dependent variable. We have binary indicators for government (SPÖ, ÖVP) and opposition parties (FPÖ, Greens, BZÖ, Team Stronach) and distinguish negative statements among the coalition partners SPÖ and ÖVP from other party pairs. We use the performance of a party in the pre-electoral polls (using the net difference in poll standings at the beginning of the campaign with the election result) and the proximity of the election (in days) as control variables. Empirical research shows that parties that are losing ground in the electoral competition employ more negative campaigning and that campaigns become increasingly negative towards the end (Damore 2002).

**Fig. 5:**
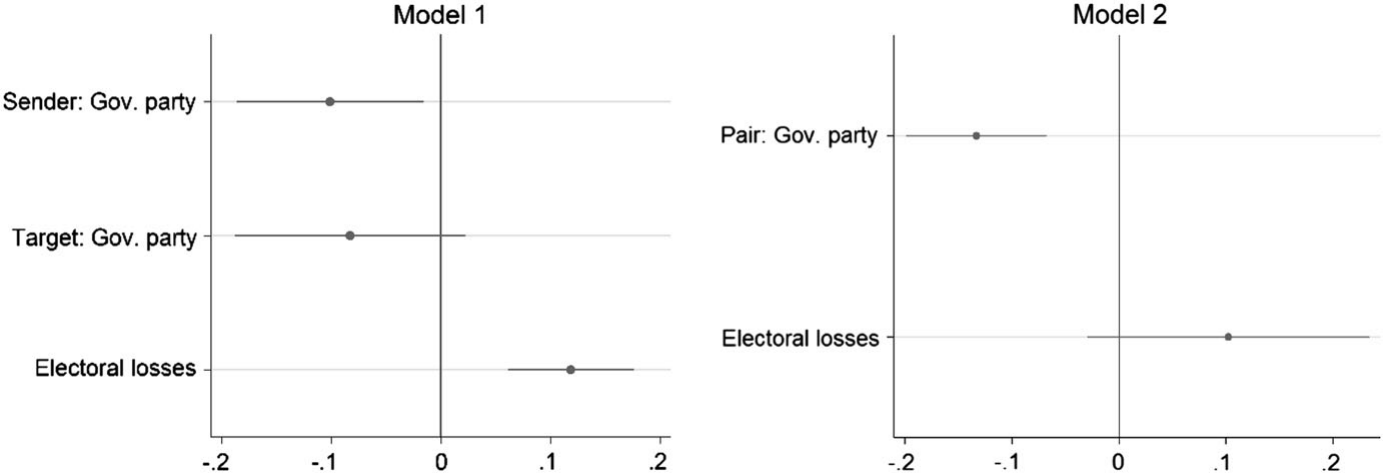
OLS regression coefficients (with 95 %-confidence intervals)

 The results in Table 3 and Fig. 5 indicate significant differences with regard to the tonality of negative campaign messages made by government and opposition parties. We also find evidence that parties refrain from using aggressive statements against their coalition partner.

**Table 3:** OLS regression of negative campaigning tonality

	Model 1	Model 2
Sender: Gov. party	−0.10*	–
	(0.04)	
Target: Gov. party	−0.08	–
	(0.05)	
Pair: Gov. party		−0.13***
		(0.03)
Electoral losses	0.12***	0.10
	(0.03)	(0.06)
Proximity of the election	−0.01	−0.01
	(0.03)	(0.03)
Constant	2.28***	2.22***
	(0.06)	(0.04)

Party fixed effects	Yes	Yes
Observations	619	619
Adjusted *R* ^2^	0.03	0.03
Log likelihood	−446.16	−445.81

Standard errors clustered across party pairs in parentheses

* *p* < 0.05, *** *p* < 0.001

Negative campaigning from a government party was ceteris paribus 0.1 units less negative than negative statements from opposition parties. Inter-government conflict was on average 0.13 units less negative than criticism exchanged between other party pairs. Parties showing bad electoral performance issued more strongly worded campaign messages, but this effect disappears in the second model. We find no escalation of negativity towards the end of the campaign.

### Media tonality in campaign reporting

The second application focusses on how the media cover politics and transmit the parties’ campaign messages. These topics deserve study because mass media are the most important source of information for voters about a specific electoral contest (Strömbäck and Kaid 2008). Our starts off from the classic finding (Galtung and Ruge 1965) that negativity is a highly important factor determining the newsworthiness of an event. A wealth of studies from the United States has established the findings of the media’s focus on negative stories or its cynical reporting on politics (Patterson 1993; Capella and Jamieson 1997; Farnsworth and Lichter 2010).

We want to know whether sentences in media reports that mention a political party or a top candidate are more critical than statements without a reference to these political actors. We use the dictionary to measure the tonality of reporting on the six parliamentary parties and their top candidates in fifteen Austrian daily newspapers.

**Fig. 6:**
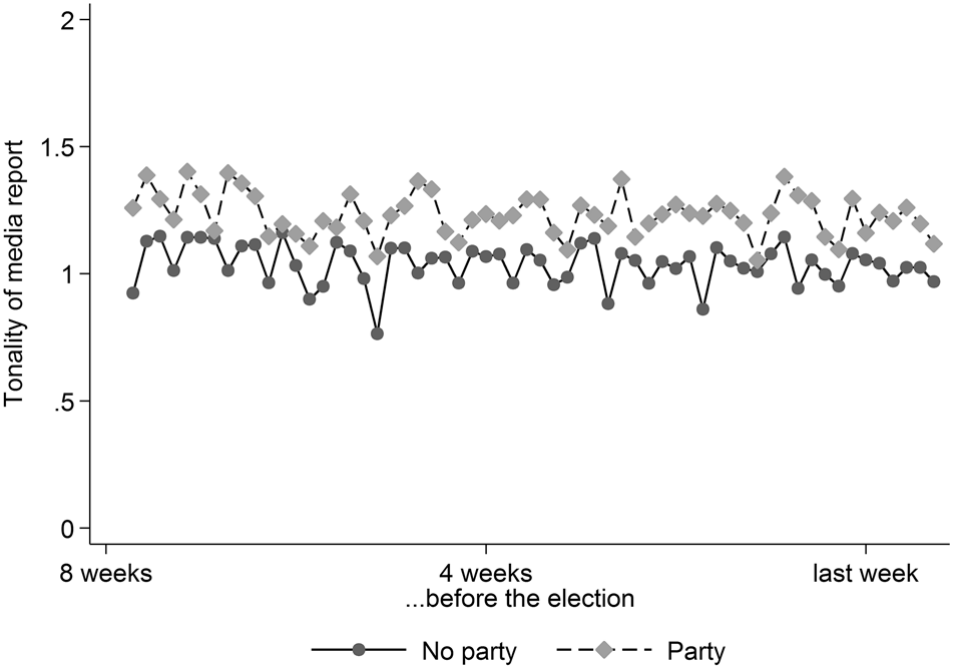
Mean tonality of campaign coverage on parties and others

The raw data consist of 15,096 news reports published during the final 8 weeks of the campaign for the national parliamentary elections of 2013. The media reports were collected as part of the Austrian National Election Study (Haselmayer et al. 2013; Kleinen-von Königslöw et al. 2013). They consist of 439,954 sentences, of which about one in five has a reference to a party or candidate. Slightly more than half (55 per cent) contain a dictionary word. For the rest we assume that the scope of the dictionary is sufficient to identify all overtly negative statements and code them as neutral statements. We compare the tonality of sentences with and without a reference to a party or candidate and find that the mean tonality of sentences with a reference is 1.23 across the fifteen print news outlets compared to 1.00 for the contrast group of statements without actor reference. It indicates that media coverage was slightly negative on average. Figure 6 shows the temporal variation in the last 6 weeks before the election. The slightly more negative tone whenever a political actor is mentioned can be clearly seen. Note that this application is purely illustrative. Whether it constitutes evidence of a critical or cynical perspective of journalists on politics (Patterson 1993; Capella and Jamieson 1997) would require further study.

## Conclusions

This article shows how to create a dictionary-based measurement procedure for negative sentiment in a language of choice that is cheap, fast, reliable and valid when compared to human coding. The English language bias of computer-based sentiment analysis constrains social scientists interested in studying textual data in other languages. For this reason, with the help of crowdcoders, we created a German political language dictionary tailored to party statements and media reports. Our results underscore that crowdcoding is a viable alternative to the use of expert coders or trained coders in the social sciences (Benoit et al. 2016). Yet, even if the costs (in terms of both, money and time) are low when compared to trained coders, they increase linearly. Thus, large-scale analyses of (comprehensive) text units clearly impose limits to manual coding.

Thus, computer-based sentiment analysis dominates the field. Its amazing pace of innovation, low costs and scalability make it a highly attractive, alternative approach. Yet, most tools for (semi-)automated text analyses were developed using English language texts. Convinced of their value social scientists frequently apply them to texts in other languages. Algorithms do not object to a language transfer as long as the strings to be processed appear well formed. Yet, prudent users should require empirical evidence on how well an automated text analysis tool operates in another language, which brings us back to the need for validation (Grimmer and Stewart 2013; Lowe and Benoit 2013).

We have empirically demonstrated the importance of using a customized dictionary. Two alternative sentiment dictionaries had substantially lower coverage and agreement with manual codings of sentiment strength. We would recommend the use of any general-purpose dictionary with caution (see already Grimmer and Stewart 2013; González-Bailón and Paltoglou 2015; Soroka et al. 2015a).

Our fine-grained dictionary-based sentiment scores move beyond a polarity classification of text. Although the sentences taken from press releases and media reports were a challenging test set our dictionary-based scores reflected the human ratings of crowdcoders and expert coders to a large degree. While the bag-of-words approach has its limitations, for example when confronted with figurative language, it performed well in the vast majority of cases.

Sentiment analysis offers many exciting avenues for innovative social science research. We have shown the usefulness of our new sentiment dictionary in two applications: negative campaigning by parties and media tone. Future research could look at the incentives for negative campaigning in multi-party systems, for example rhetorical interaction between potential coalition partners (e.g., Walter and Van der Brug 2013), and study the effects of campaign tonality on post-electoral government formation.

Parallel sentiment analyses of campaigns and its media coverage allow an empirical assessment whether parties can attract media attention through negative campaign messages as hypothesized by Geer (2006). Comparing the tonality of party campaign messages and their news coverage enables testing the presumed ‘negativity bias’ of the news media (Patterson 1993; Capella and Jamieson 1997; Farnsworth and Lichter 2010). Sentiment analyses can also add to our understanding of the effects of negative campaigning and media negativity on voting behavior. Studying how voters react to variation in campaign tonality provides new contributions to the debate on potential benefits from negative campaigning with regard to political knowledge and turnout (e.g., Lau and Pomper 2004; Geer 2006).

Sentiment analysis can also contribute to other research topics at the intersection of communication science and political science such as the study of public opinion and political polarization (e.g., Monroe et al. 2008; Hopkins and King 2010; González-Bailón and Paltoglou 2015). Parliamentary debates, party manifestos, blogs and social media platforms provide rich data sources for sentiment analyses. We have shown how to create and use a dictionary for large-scale sentiment analyses. There are no limits in using the same or similar procedures to create a customized dictionary for other research areas.

Finally, we foresee sentiment analyses with multi-language dictionaries. Debate transcripts from the European Parliament or the United Nations General Assembly provide multi-lingual textual data. Comparative sentiment analyses can submit textual data in several languages to crowdcoding or use tools for automated text translation. We have identified several research topics that may benefit from fine-grained sentiment analyses and have described our procedure for German language texts. Hopefully, we convinced readers that undertaking similar analyses with textual data in a language of choice is worthwhile and doable.

## Figures and Tables

**Fig. 7:**
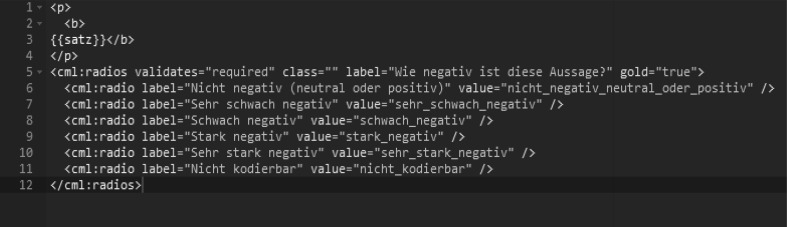
CrowdFlower Markup Language for the coding of sentence tonality

**Fig. 8:**
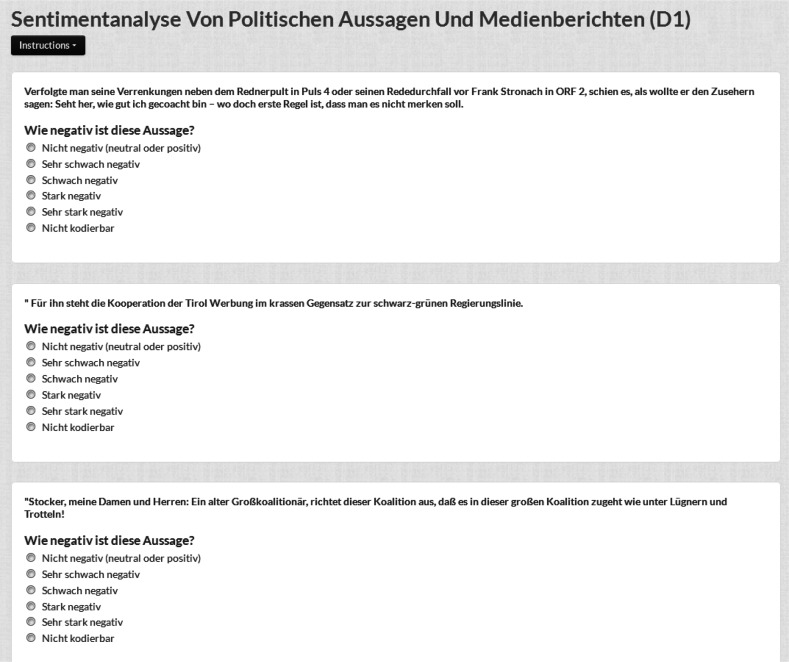
Screenshot of the CrowdFlower coding interface

## Appendix

### Appendix 1: Text preprocessing

Automated text analysis requires text preprocessing. We found the performance of current natural language processing tools for lemmatization and part-of-speech tagging of separable verbs, compounds, dialectal variations, and the frequent neologisms in German political and media texts to be rather unsatisfactory and manually cleaned incorrect lemmas. Given imperfect lemmatization, we allow for imperfect word matching to increase the number of matches when applying the dictionary to new texts. Stemming (Porter 1980) is common in English language applications as an alternative to lemmatization, but stemming worked worse in our applications and produced the lowest correlation with manual coding (see Table 4).

**Table 4:** Effects of text preprocessing on correlations with manual coding

	Stemming	Lemma-tizing	Stemming, part-of-speech tagging, stop word and named entity deletion	Lemmatizing, part-of-speech tagging, stop word and named entity deletion
Coverage (%)	100	99	99	84
Pearson correlation with manual coding	0.13	0.34	0.42	0.65

n = 200

A combination of lemmatization, part-of-speech-tagging and deletion of stop words and named entities gave the best result (Schmidt 1994; Quasthoff et al. 2006; Faruqi and Pado 2010; Steinberger Ralf et al. 2011; Benikova et al. 2014).

### Appendix 2: Effect of different aggregation rules

Table 5 shows how different aggregation rules for word and sentence scores such as mean-of-means for word scores (see Benoit et al. 2016) and selecting the most negative word as sentence score (Thelwall et al. 2012) as well as accounting for intensifier and negation words (Taboada et al. 2011; Thelwall et al. 2012) affect the correlations with manual expert coding.

**Table 5:** Effect of aggregation rules on correlations with expert coding

Word score	Majority voting				Mean			
Sentence score	Mean	Max	Mean	Max	Mean	Max	Mean	Max
Booster and negation words	No	No	Yes	Yes	No	No	Yes	Yes
Pearson correlation with expert coding	0.38	0.39	0.39	0.38	0.49	0.58	0.54	0.65

n = 168 dictionary-based scores for 200 test sentences

We obtained the highest correlation using means for calculating word scores, choosing the most negative word as sentence score and by accounting for intensifier and negation words.

### Appendix 3: Validation with a larger sample and single expert coding

An additional validation test uses a larger sample (n = 755) of negative statements in party press releases from the same election campaign which were coded by only one of the authors. The dictionary’s coverage was 82 % and the correlation between manual and dictionary based sentiment coding 0.63. We validate the results of our regression model by re-running the same models as in Table 3 with the single expert coding as dependent variable. Table 6 shows identical signs of coefficients for all effects compared with the dictionary-based sentiment scores. The automated scoring procedure tends to show weaker effect sizes due to the two-step-mean aggregation. We conclude that our dictionary-based scoring produces valid sentiment estimates at the level of analysis that are more conservative than results based on manual coding. Rerunning the regression after assigning zero negativity to sentences that had no matching dictionary word (n = 136) did not change these results (not reported).

**Table 6:** OLS regression of the tonality of party press releases using dictionary-based and manually coded scores

	Model 1 (dictionary)	Model 1 (expert)	Model 2 (dictionary)	Model 2 (expert)
Sender: Gov. party	−0.10*	−0.18^+^	–	–
	(0.06)	(0.10)		
Target: Gov. party	−0.08	−0.22*	–	–
	(0.05)	(0.09)		
Pair: Gov. party			−0.13***	−0.40***
			(0.03)	(0.05)
Electoral losses	0.12***	0.23***	0.10	0.04
	(0.03)	(0.06)	(0.06)	(0.05)
Closeness to the election	−0.01	0.01	−0.01	0.01
	(0.03)	(0.06)	(0.03)	(0.06)
Constant	2.28***	2.57***	2.22***	2.53***
	(0.03)	(0.11)	(0.04)	(0.06)

Party fixed effects	Yes	Yes	Yes	Yes
Observations	619	755	619	755
Adjusted *R* ^2^	0.03	0.04	0.03	0.04
Log likelihood	−446.16	−954.03	−445.81	−952.06

Standard errors clustered across party pairs in parentheses. Differences in the number of observations are due to sentence with no matching dictionary words

^+^ *p* < 0.1, * *p* < 0.05, *** *p* < 0.001

### Appendix 4: CrowdFlower coding jobs

Table 7 provides information on the online coding jobs.

**Table 7:** CrowdFlower coding jobs

Job	Date launched	Date completed	Sentences	Valid codings (incl. test questions)	Sentences per task	Payment per task	Codings per unit	Number of coders	Cost	Countries
1	23.01.2014	23.01.2014	50	225	5	5	3	21	US$ 2.45	GER, AUT
2	26.03.2014	02.04.2014	4875	48,779	10	10	10	188	US$ 558.00	GER, AUT
3	26.03.2014	01.04.2014	4875	48,826	10	10	10	182	US$ 564.30	GER, AUT
4	05.04.2014	18.04.2014	490	4968	10	10	10	40	US$ 59.40	AUT
5	21.10.2014	22.10.2014	100	1000	5	5	10	35	US$ 18.18	GER, AUT
6	12.11.2014	18.11.2014	5452	54,618	10	10	10	276	US$ 872.88	GER, AUT
7	11.12.2014	12.12.2014	234	2379	10	10	15	44	US$ 63.54	GER, AUT
8	18.03.2015	23.03.2015	200	2026	5	5	10	243	US$ 59.48	GER, AUT

Coding speed and costs depend on a variety of factors including geographical restrictions, requested skills, experience or performance levels, the number of codings per coder and the ratio of “missed” test questions. The crowdcoding platform charges a percentage due of the ‘transaction costs’ for a coding task. The dues were set at 10 % at the start, and following a raise, 20 % for the last coding jobs

### Appendix 5: Coding instructions (translation)

The following coding instructions were pretested by colleagues, student assistants and a few online coders.

How negative are these statements?

#### What is this about?

We present you sentences from political and media texts. Many, though not all, of these sentences include direct or indirect criticism, allegations or attacks.

#### Task

Please read each sentence carefully and decide, whether it includes a positive, neutral or negative statement. In a second step, we ask you to rate the intensity of the statement using the following scale:

- Not negative (neutral or positive)
- Very weakly negative
- Weakly negative
- Strongly negative
- Very strongly negative
- Not codable

#### What should you consider?

Only rate the actual content of the text! Stay impartial, your personal preferences towards persons or organizations should not influence your coding decisions.

#### Not negative

A sentence should be coded as “not negative” if it contains a neutral or positive statement.

Example “not negative”:

“I serve the Austrian citizens with passion and commitment.”

#### Not codable

A sentence is “not codable” if it is incomprehensible or if it does not make any sense to you.

Some sentences may be incomplete, as they have been processed automatically. As long as you are able to purposefully decide, whether they are positive, neutral or negative, we ask you to rate them anyhow.

Example “not codable”:

“Ic$ %$#* we retain %, that & %§”

#### Negative

Negative sentences contain direct or indirect criticism, allegations or attacks in varying intensity.

Examples with increasing negativity:

“We demand that the government finally delivers a better job!”

“These are bad actions, which come at the expense of the population.”

“This minister promotes corruption and consciously dupes the people.”

“This is a scam on all of us: the dishonesty of these politicians stinks to high heavens.”

#### Special case: sentences containing specific coding instructions

Some sentences may contain instructions, asking you to choose a specific category. In such cases, you should ignore all other textual information and directly follow the instructions.

Example:

“The government has failed to address these issues in the past legislative term. Please ignore the previous part of the text and code this unit as “not codable”.

In case of any question regarding the coding process or if you would like to provide us with feedback, please send us an E-Mail: crowdsourcing@autnes.at.

Thank you for your contribution!

### Appendix 6: CrowdFlower task script

CrowdFlower jobs have to be designed in CrowdFlower Markup Language (CML), which is entirely based on HTML, but contains a small set of special features (e.g. to link data). Its implementation for simple coding tasks, such as the annotation of sentiment strength is very easy (Fig. 7):

### Appendix 7: CrowdFlower coding task

Figure 8 shows a screenshot of the coding task as presented to our crowdcoders. Instructions (see Appendix 3) are hidden for a better readability. One page usually contains five sentences along with the question “How negative is this statement?” and the labelled coding options.

## Footnotes

1
https://homepage.univie.ac.at/martin.haselmayer/.

2
http://provalisresearch.com/products/content-analysis-software/wordstat-dictionary/sentiment-dictionaries/.

3 The tasks carried out on crowdsourcing platforms extend far beyond text coding. We use the term crowdcoding for content analysis tasks.

4 Press releases and media reports were collected as part of the Austrian National Election Survey (AUTNES). The parliamentary debates are available online at the Parliament’s Website (https://www.parlament.gv.at).

5 The target sample size corresponded to a budget limit of about 2000 Euros (roughly 2200 US-dollars) for coding, based on estimated task duration and payment of local minimum wages. After deleting duplicate and incomplete sentences from the automatic text preprocessing, the final sample size was 12,713 sentences.

6 An accuracy threshold of 75 % means three of the four initial test questions have to be correct. Including “uncodable” a coder has six options with two accepted as correct. The probability of passing by guessing then is $\left( {\frac{2}{6}} \right)^{3}$ = 0.04.

7 We test other aggregation algorithms such as mean scores of all words, which resulted in a lower correlation with the expert coding. See the Appendix with the results of different aggregation rules.

8 We provide an extended validation with a larger set of sentences coded by one of the authors in the Appendix, where we also report on effects of text preprocessing. The binary predictive quality of the dictionary (negative vs. not negative), based on a standard confusion matrix (e.g. Davis and Goadrich 2006; Fawcett 2006) results in an accuracy of 0.85, the F-score is 0.91.

9 SentiWS (Remus et al. 2010) contains positive and negative words along with word flexion (conjugation/declination). The sentiment score provided by the dictionary indicates the probability of the word appearing in a positive or negative context. The sentiment dictionary created by Momtazi (2012) contains positive and negative words and word stems, as well as valence shifters and negation words.
